# Sebaceoma on the scalp simulating a malignant pigmented neoplasia^☆^^☆☆^

**DOI:** 10.1016/j.abd.2019.09.007

**Published:** 2019-09-30

**Authors:** Bárbara Catojo Poggi, Daniel Fernandes Melo, Juliana Marques da Costa, Maria Auxiliadora Jeunon Sousa

**Affiliations:** a Department of Dermatology, Hospital Naval Marcílio Dias, Rio de Janeiro, RJ, Brazil; b Postgraduate Program in Medical Sciences, Hospital Universitário Pedro Ernesto, Universidade do Estado do Rio de Janeiro, Rio de Janeiro, RJ, Brazil; c Clinic of Oncologic Dermatology and Dermatoscopy, Department of Dermatology, Hospital Naval Marcílio Dia, Rio de Janeiro, RJ, Brazil; d ID – Investigação em Dermatologia, Rio de Janeiro, RJ, Brazil

**Keywords:** Adenoma, Dermoscopy, Histology, Melanoma, Neoplasms, Sebaceous gland neoplasms, Sebaceous glands, Skin neoplasms, Trichoscopy

## Abstract

The correct identification of pigmented nodular lesions of the scalp is often challenging. Despite the importance of clinical patterns and dermoscopy, important adjuvant tools that are usually helpful, their interpretation sometimes is not clear-cut. Here, the authors discuss a case of sebaceoma mimicking a malignant pigmented neoplasia, with conclusive histopathology.

## Introduction

Diagnosis of pigmented nodular lesions of the scalp is often challenging. Clinical findings and dermoscopy are important in the suspicion of malignant melanocytic lesions; nevertheless, histopathology maintains its fundamental role in diagnostic conclusion, since other lesions can mimic them.

## Case report

**Figure 1:**
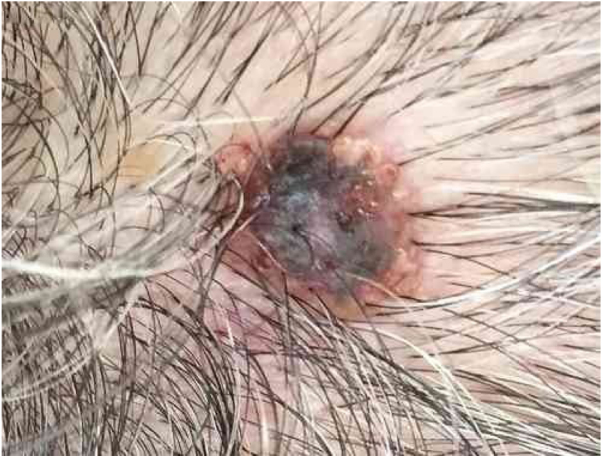
Blackened nodule upon erythematous base with central crust at the apex of the scalp.

**Figure 2:**
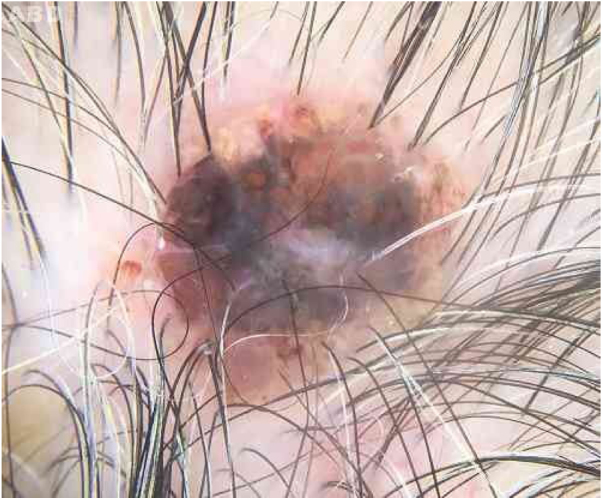
Hematic crust and peripheral red-milky area with poorly defined globules on dermoscopy.

An 80-year-old female patient, Fitzpatrick phototype II, with a history of endometrial cancer, developed a black, nodular, and asymptomatic lesion with erythematous base and central crust on the vertex of her scalp (Fig. 1). Dermoscopy showed a yellowish erythematous area, yellow globules, hematic crust, a red-milky area, and polymorphic vessels, suggesting seborrheic keratosis, an adnexal tumor, or a traumatized melanocytic nevus (Fig. 2). The presence of a whitish veil, a bright white area, asymmetric follicular openings, and rhomboidal structures did not allow exclusion of cutaneous melanoma (Fig. 2). An excisional biopsy was performed and histopathological examination evidenced a circumscribed proliferation of large basaloid cell masses and sebaceous cells, with the diagnostic conclusion of sebaceoma.

## Discussion

Sebaceoma has been called sebomatrixoma or sebaceous epithelioma, when the use of the term epithelioma suggested malignancy. Classified by Troy and Ackerman in 1984 as a benign neoplasm with sebaceous differentiation, it more often affects women, with predominance in the eighth decade of life.^1^

Clinically, it appears as a yellowish or orange, solitary, or rarely multiple hemispheric exophytic tumor located in the seborrheic areas of the body, especially on the scalp.^1^, ^2^

Dermoscopy of sebaceoma may present an amorphous yellowish-erythematous area with or without ulcerations, with centripetally branched arboriform vessels. The amorphous yellowish-erythematous area may be an important finding suggesting the sebaceous etiology of the lesion.^3^, ^4^

Several benign adnexal tumors may present as a single nonspecific lesion; therefore, histopathological examination is fundamental for definitive diagnosis. There are tumors for which no malignancy is suspected because they lie more deeply in the dermis and may resemble cysts. However, on several occasions, benign tumors are connected to the epidermis or touch it, with the possibility that traumatic ulcerations and thereby malignancies can be mimicked.^5^, ^6^

**Figure 3:**
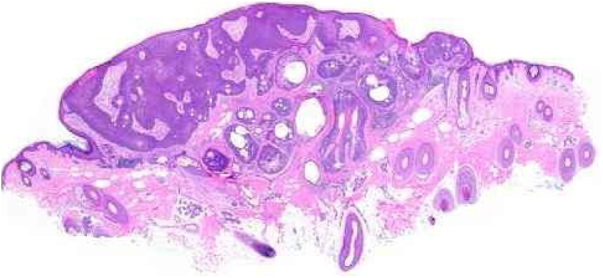
Pathological features of sebaceoma. Regularly contoured epithelial neoplasia arranged in “v” with the apex pointing toward the depth. There is acanthosis on the left and predominance of dermal masses on the right, conferring intrinsic asymmetry, an unusual characteristic for this type of proliferation. The masses have regular shapes and sizes, are predominantly rounded or oval, and are immersed in collagenized stroma. In this panoramic magnification, it is already possible to perceive clusters of epithelial cells of pale cytoplasm permeating the masses; this is representative of mature sebocytes (hematoxylin and eosin, ×20).

Benign and malignant tumors are identified by the type of differentiation they exhibit, the remnants of their origin cells, although malignant tumors lack the richness of findings that benign variants show. Regarding sebaceous tumors, the signs of differentiation are sebaceous cells and sebaceous ducts (Fig. 3).^5^

Sebaceous glands are composed of several lobes leading to a duct connected to the hair follicle. There is a peripheral single row of undifferentiated cells and, toward the center, cells with increasing degrees of differentiation by fat synthesis until the well-differentiated sebocytes in which the nucleus is indented by various depressions caused by large fat vacuoles. Near the duct, the sebaceous cells lose their nuclei, and thus the sebaceous secretion known as holocrine is eliminated.

Sebaceomas are constituted by masses located in the dermis, connected or unconnected to the epidermis, with histological architecture that suggests benignity: rounded contours, a greater vertical axis, and symmetry, different from the sebaceous carcinomas despite the possible presence of mitosis (Fig. 3). They are composed of undifferentiated cells and cells with different degrees of sebaceous differentiation. The absence of peripheral palisade and clefts between the aggregates and the stroma distinguish them from basal cell carcinomas with sebaceous differentiation.^5^, ^6^

What differentiate the sebaceous adenoma from sebaceoma are the cellular arrangement and the proportion of undifferentiated cells, more numerous in sebaceoma. In sebaceous adenoma, the arrangement of the latter in the periphery of the aggregates and in those with some degree of differentiation in the central portion resembles the normal sebaceous gland, facilitating its identification. However, unlike the normal gland, there are several layers of peripheral undifferentiated cells. This proliferation replaces the epidermis and its secretion is released straight to the surface, instead of draining into the follicular unit through the sebaceous duct where the sebaceous secretion of the normal gland reaches the surface.

In sebaceoma, with the two types of cells arranged in a disordered way, the search for sebocytes with more advanced differentiation – and they may be sparse – is necessary. In contrast, the sebaceous ducts, absent in the adenomas, are present in the sebaceomas and serve as a clue for the active search of sebocytes.

Although the sebaceous adenoma has the architectural and cytological characteristics closest to the normal gland, some authors considered it as a second type of sebaceous carcinoma due to presence of cell stacking and mitotic figures in undifferentiated peripheral cells.^6^, ^7^

**Figure 4:**
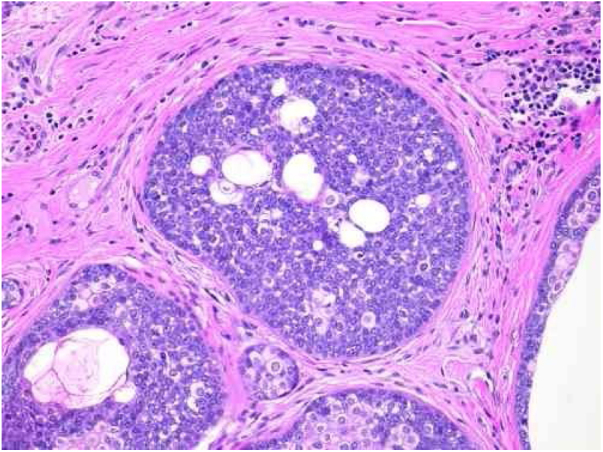
Pathological features of sebaceoma. Detail of epithelial massive arranged in the dermis. The predominance of vesicle-like epithelial cells and scarce cytoplasm (immature sebocytes) can be noted, which are less permeated by pale cytoplasm cells, containing lipid vacuoles, some with indented nuclei (mature sebocytes), randomly arranged and associated with ducts. There is absence of palisade in the periphery of the masses (hematoxylin and eosin, ×200).

The distinction between benign and malignant neoplasms considers the degree of differentiation, with the malignant neoplasms having lower degrees, since the cellular apparatus is geared toward cell division rather than synthesizing substances involved in differentiation. Advanced differentiation and malignity are opposing concepts (Fig. 4).^6^

It is possible to suppose that the secretion of sebaceous adenomas, being freely eliminated on the surface, does not suffer the effect of a retrograde compression, as should occur with the passage of sebum through a narrow duct in the normal gland. It is a speculation to conclude that secretion retained until its elimination is a factor that inhibits cell division and that its absence in sebaceous adenomas favors cell proliferation. It is important to remember that in the bulbs of anagenic follicles, producing a hair shaft continuously at a reasonable rate, cell stacking and mitosis also occur.

**Figure 5:**
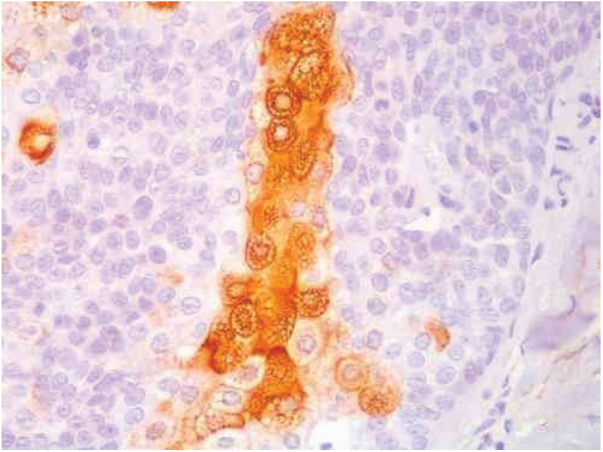
Immunohistochemical staining of the skin biopsy: positivity in the immunohistochemical reaction with the anti-EMA antibody in a cytoplasmic reticular pattern around the lipid vacuoles (original magnification of ×400).

Immunohistochemical study in sebaceoma shows multifocal positivity of the neoplastic cells with anti-CK7 and anti-EMA antibodies, and negativity in reactions with anti-CK20 and anti-BerEp4 antibodies (Fig. 5).

Presence of neoplasms with sebaceous differentiation or multiple keratoacanthomas may be revealing of Muir-Torre Syndrome, which also has visceral adenocarcinomas located mainly in the gastrointestinal and genitourinary tracts, endometrium, and larynx. It predominantly affects men in the fifth decade of life, and clinical screening is mandatory, as in the case of the patient in question.^8^, ^9^

## Final considerations

The diagnosis of pigmented nodular lesions of the scalp is challenging. Dermoscopy is an excellent tool for the definition of these cases, having well-established standards for the diagnosis of neoplasms, pigmented or not. However, as in the case reported, the histopathological study remains the gold standard for diagnosis, and was fundamental for the elucidation of the sebaceous nature described above.

## Funding

None declared.

## Author's contribution

Bárbara Catojo Poggi: Elaboration and writing of the manuscript; obtaining, analyzing and interpreting the data.

Daniel Fernandes Melo: Approval of the final version of the manuscript; conception and planning of the study; elaboration and writing of the manuscript; intellectual participation in propaedeutic and/or therapeutic conduct of the cases studied; critical review of the manuscript.

Juliana Marques da Costa: Approval of the final version of the manuscript; elaboration and writing of the manuscript; intellectual participation in propaedeutic and/or therapeutic conduct of the cases studied; critical review of the manuscript.

Maria Auxiliadora Jeunon Sousa: Approval of the final version of the manuscript; elaboration and writing of the manuscript; effective participation in research orientation; intellectual participation in propaedeutic and/or therapeutic conduct of t.he cases studied; critical review of the literature; critical review of the manuscript.

## Conflicts of interest

The authors declare no conflicts of interest.
